# Zika virus-induced hyper excitation precedes death of mouse primary neuron

**DOI:** 10.1186/s12985-018-0989-4

**Published:** 2018-04-27

**Authors:** Julie Gaburro, Asim Bhatti, Vinod Sundaramoorthy, Megan Dearnley, Diane Green, Saeid Nahavandi, Prasad N. Paradkar, Jean-Bernard Duchemin

**Affiliations:** 1 CSIRO Health and Biosecurity, Australian Animal Health Laboratory, Geelong, Australia; 2 Institute for Intelligent Systems Research and Innovation (IISRI), Deakin University, Geelong, Australia

**Keywords:** Zika, Primary neuron, Microelectrode array, Excitotoxicity, Hyper excitation, Spike, Glutamate

## Abstract

**Background:**

Zika virus infection in new born is linked to congenital syndromes, especially microcephaly. Studies have shown that these neuropathies are the result of significant death of neuronal progenitor cells in the central nervous system of the embryo, targeted by the virus. Although cell death via apoptosis is well acknowledged, little is known about possible pathogenic cellular mechanisms triggering cell death in neurons.

**Methods:**

We used in vitro embryonic mouse primary neuron cultures to study possible upstream cellular mechanisms of cell death. Neuronal networks were grown on microelectrode array and electrical activity was recorded at different times post Zika virus infection. In addition to this method, we used confocal microscopy and Q-PCR techniques to observe morphological and molecular changes after infection.

**Results:**

Zika virus infection of mouse primary neurons triggers an early spiking excitation of neuron cultures, followed by dramatic loss of this activity. Using NMDA receptor antagonist, we show that this excitotoxicity mechanism, likely via glutamate, could also contribute to the observed nervous system defects in human embryos and could open new perspective regarding the causes of adult neuropathies.

**Conclusions:**

This model of excitotoxicity, in the context of neurotropic virus infection, highlights the significance of neuronal activity recording with microelectrode array and possibility of more than one lethal mechanism after Zika virus infection in the nervous system.

**Electronic supplementary material:**

The online version of this article (10.1186/s12985-018-0989-4) contains supplementary material, which is available to authorized users.

## Background

Since its identification in 1947, Zika virus (ZIKV) has been mostly associated with asymptomatic infections in humans [1]. However, the recent ZIKV outbreak in Brazil revealed a strong link between ZIKV infections and neuropathologies of the central nervous system (CNS), especially in newborns [2]. Congenital syndrome with microcephaly associated with infection in South America has been described [3, 4] and largely studied using in vivo models [5–8]. Within the embryo’s brain, Zika virus has a strong preference for radial glial progenitor cells [9, 10], which causes defects in cell proliferation and increase in neuronal progenitor death [11]. These studies have shown that human neurons are susceptible to ZIKV, providing a potential cause for microcephaly observed in human and mouse fetuses [12]. Zika virus neurovirulence has also been studied in vitro with human pluripotent stem cell (hPSC)-derived neural progenitor cells and brain organoids [13–15] and has provided some insights into clinical pathology, such as microcephaly.

Although ZIKV seems to mainly target neural progenitors in embryo during the early phase of brain development, worrying questions remained unanswered regarding the infection at later stage of pregnancy and delayed pathology during childhood or adulthood. Besides congenital disorders, Zika virus infection has also been linked to Guillain-Barre syndrome (GBS) [16–19], which affects the peripheral nervous system (PNS), as well as recent records of encephalitis [20] and myelitis [21]. Although ZIKV seems to mainly target neural progenitors in the developing brain, it has also been found in some areas of the adult brain [22].

Cell death following ZIKV infection is mostly due to apoptosis with activation of caspase 3, throughout the brain [23] of postnatal mice infected intra-cranially with ZIKV. However, partial overlap of staining between cleaved caspase 3 and ZIKV infected cells suggest that the infection may induce apoptosis and cell death through a non-cell autonomous mechanism. Involvement of innate immune system via TLR3 [24] as well as of N-Methyl-D aspartic acid Receptor (NMDA-R) [25] have been proposed upstream cellular mechanisms of cell death for ZIKV associated neuronal loss. Costa et al. suggested neuronal excitotoxicity, mediated by excessive or prolonged activation of excitatory amino acid receptors, as cause of ZIKV-induced neuronal death. Glutamate-induced influx of ions is mediated predominantly by NMDA-R in neuron cultures, and treatment with NMDA-R antagonists protects the cells from glutamate-induced death [26]. This mechanism has been previously shown to be involved in the pathogenesis of ischemic brain injury, epilepsy, and neurodegenerative diseases [27] via glutamate, which is known to trigger neuronal death when present in excess quantities [28]. In Sindbis virus infected neurons, glutamate excitotoxicity was shown to be an important mediator of early virus-induced neuronal death [29]. Altogether, these results imply that ZIKV could induce neuronal cell death, both directly and indirectly, through multiple pathways.

Here we aim to elucidate the possible mechanism(s) of neuronal cell death after ZIKV infection by studying temporal electrical activity of mouse primary neuronal network using Microelectrode array (MEA). Along with electrophysiological data, we also followed the viral replication dynamics in primary mouse neuron cultures as well as neuronal morphology using confocal microscopy. Finally, mRNA levels of glutamate and GABA neurotransmitters were analyzed, confirming the involvement of glutamate in ZIKV-induced neurotoxicity in the mouse embryonic neuron culture model.

## Methods

### Viruses’ preparation and strain

All experimental assays were done using Zika virus (ZIKV) human isolate from Cambodia 2010 (Genbank KU955593 [30]) grown in Vero cells. Dengue virus type 2 ET300 (DENV2) isolated in Australia from a soldier returning from East Timor was passaged 7 times in C6–36 cell line.

### Primary neurons dissociation, culture and infection

Cortical embryo primary neurons from mouse, *Mus musculus*, were prepared by the tissue culture laboratory at the Australian Animal Health Laboratory (AAHL-CSIRO) under the permit AEC number 1686. Whole brains were extracted from C57BL/B6 mouse embryos at embryonic day 15 (E15) after decapitation. In aseptically conditions to avoid contamination, cortical neurons from embryos were removed from the brain by gently removing the meninges in cold dissection medium Hibernate (Gibco®). Isolated cortex hemispheres were then treated with 5 mg/ml Trypsin and 0.75 mg/ml DNAseI in Minimal Essential Medium (Gibco®) for 5–10 min at 37 degrees Celsius to perform enzymatic dissociation. After three washes with the dissection medium, mechanical dissociation was performed by 10 passages through a 10 ml glass pipette. The cell suspension was then centrifuged at 100×g for 5 min, and pellet was re-suspended in supplemented Neurobasal® Medium (ThermoFisher®) culture medium.

Neuronal cultures for confocal microscopy assays, TCID_50_ and RT-PCR: Tissue culture plate wells and coverslips for confocal microscopy were pre-coated with 100 μl of polyethyleneimine (PEI, Sigma, 0.05% in Borate-buffered solution) at room temperature (RT) for 30 min, rinsed 3 times with Tissue culture treated water and let to dry out in the Biosafety Cabinet class II (BSCII). Cells were plated in a 24-well plate with 25 μl from a stock solution of 3.10^6^ cells/ml. The plate was placed in incubator for half an hour for cells to adhere before adding 1 ml of media. Cells were cultured at 37 °C with 5% CO2. Wells were topped up with Neurobasal media for mouse neurons completed with B27 Supplement, Glutamax and Gentamycin.

Neuronal cultures on Microelectrodes array: The method modified from Hales et al. [31] was used. Microelectrodes array (MEA) were pre-coated before cell seeding. The coating consists of 100 μl PEI at RT for 30 min, followed by TC-water rinses (3 times); laminin (0.02 mg/ml, Sigma L-2020) is finally added for 20 min at 37C and 5% CO2, just before plating. Cells were plated in the center of the MEAs at 7.5.10^4^ cells per device, corresponding to a volume of 25 μl from a 3.10^6^ cells/ml stock cell solution. After half an hour in the respective incubator for cells to adhere to the bottom of the MEA well, 1 ml of cell media was added. Half of the culture medium was changed every 3–4 days excluding the day before recording.

Primary neuron culture infection: All primary neuron cultures were infected with the same Multiplicity of Infection (MOI) of 0.2 for ZIKV as well as DENV-2 at 7 days of in vitro (div) culture, for network maturation. *Mus musculus* cultures with ZIKV passaged in VERO cells. Neuron cultures were also infected with DENV-2 as viral control. For infection of 24-well plate culture, the whole media was removed and replaced with 1 ml of virus dilution solution for an MOI of 0.2. For infection on MEAs, virus particles were mixed in 300 μl of media and added into the MEA well. After one hour the infected media was removed and wells were rinsed with 300 μl of clean media. Finally, each well was topped up with fresh media.

### Viral titration with TCID_50_

For virus dynamics titration, 1 ml of supernatant from neuron cultures was sampled in triplicates at 24, 48 and 72 h post infection (hpi) and used for TCID_50_ assays.

Viral titers at the different time points were determined by end point titrations (TCID_50_) in VERO monolayer cell cultures. In a 96-wells TC plate, seven 10-fold dilutions of sampled supernatants were used onto Vero cells in triplicates at 37 °C with 5% CO2. Each plate was screened under inverted microscope for cytopathogenic effect at 3 and 5 days post infection and to determine TCID_50_ using Spearmann-Karber calculation method [32].

### Quantitative real time RT-PCR (Q-PCR)

For gene expression quantification, total RNA was collected from cell culture at 12, 24, 48 and 72 hpi. Media was discarded and replaced with 200 μl fresh media. Cells were removed from the bottom by gentle scraping and vigorous pipetting. Total RNA was extracted using RNeasy Plus Mini Kit (Qiagen Sciences, Maryland, MA). cDNA was prepared using random hexamers and Superscript-III reverse transcriptase (Thermo Fisher Scientific Inc. Australia) as per manufacturer’s protocol. Real-time PCR assay was performed using the SYBR® Premix Ex Taq™ II (Takara-Bio Inc., China) and running on a QuantStudio™ 6 Flex Real Time PCR System (Applied Biosystems). Forward and reverse primers of individual targeted gene are given in Additional file 1: Table S1. Settings are: 95 °C for 30 s, followed by 45 cycles of 95 °C for 5 s, 55 °C for 40 s, followed by melt-curve stage. The 2^ΔΔCt^ values were calculated at each time point for each gene as the fold-increase over uninfected control at the same time point. Samples are made in triplicates for each value.

### Immunofluorescence (IF)

Samples preparation and confocal microscopy: At different time points after infection (0, 1, 2, 3 and 7 days post infection or dpi), primary neuron cultures grown on coverslips were fixed by adding 300 μl of 4% paraformaldehyde in 0.05 M Phosphate Buffered Saline (PBSA) for 20 min under gently shaking. The coverslips were washed gently three times for 5 min using 1 ml of PBSA. Cells were permeabilized with 1 ml of 0.1% Triton X-100 (Sigma-Aldrich) in PBSA for 10 min and rinsed three times in PBSA. Non-specific binding was blocked with 0.5% BSA in PBSA for 30 min. Primary antibodies were diluted in 0.5% BSA in PBSA and incubated for 1 h at room temperature. The following primary antibodies were used: DAPI (32,670, Sigma-Aldrich), 1:200 guinea pig anti NeuN (266,004, Synaptic System), 1:500 rabbit anti Synapsin 1/2 (106,002 Synaptic System), 1:1000 chicken anti MAP2 (ab5392, ABCAM), 1:500 rabbit anti GFAP (ab7260, ABCAM), 1:200 human 4G2 anti pan-flavivirus (ab00230–10.0, Focus Bioscience). Coverslips were washed three times with PBSA for 5 min. Coverslips were then incubated in species-specific secondary antibodies diluted in 5% BSA in PBSA for 1 h at room temperature, followed by two washes of 5 min with PBSA. Coverslips were then washed twice with water and counterstain nuclei with DAPI for 10 min, before a final wash in water and mounted carefully on glass slides. The slides were observed with a 63 × objective (with oil) using a Leica SP5 confocal microscope for signal quantification and a ZEISS LSM 800 confocal microscope for virus detection in the cultures. For the number of coverslips and images taken per treatment and condition to quantify MAP2, Synapsin, NeuN and DAPI antibody signals, see Additional file 2: Table S2.

Pictures data analysis: To quantify antibody signals, raw images were extracted with LAS AF Lite software (Leica Microsystems, Germany) in single channel format for analysis. Image analysis is done with ImageJ software [33]. Images were first converted into 16-bits format with default threshold (dark background, B&W parameters). Results were normalized with the number of cells by count of DAPI particles. For DAPI counting, the option “Watershed” was applied before analysis to avoid counting merged particles. The Plugin “Analyze particles” was then used with the parameters “0.00–1.00” for circularity and “100-Inf” for size pixel.

The same type of analysis was done for NeuN counting. For Synapsin, the signal was automatically calculated by the “Measure”, and “Raw integrated density” options. For MAP2 analysis, the same processing of the images was done by converting into 16-bits and applying a default threshold. The signal of the antibody was automatically calculated by the “Measure” and “mean gray value” options.

The presence of the viruses in the primary neuron cultures was assessed by using the ZEN software from Zeiss microscopy, with the Z-stack capture option. An optical section area was captured every 0.34 μm on a total section distance of 6.8 μm. The final image was generated with the maximum intensity projection from the Extended Focus module to obtain a depth of field from the previously acquired Z-stacks.

### Data acquisition with microelectrode array

Recording material: Microelectrodes Array (MEA) were used for electrophysiological recording of neuronal networks (60MEA 200/30iR-Ti-gr, Multi-Channel Systems, Reutlingen, Germany). The glass devices consist of 59 TiN/SiN planar round electrodes (10–30 μm diameter; 100–200 μm center-to-center inter-electrode intervals) arranged in a square grid without corners. A single larger electrode served as reference ground electrode, replacing one recording electrode. Online cellular activity was recorded using the MEA60inv System (Multi Channel Systems, Reutlingen, Germany). Action potential was recorded at each electrode sampled at 10 kHz. MC_Rack software (Multi Channel Systems), installed on the acquisition computer, allowed files acquisition. Data analysis was performed off-line using MC_Rack software by Multi Channel Systems and NeuroSigX software developed by researchers at Institute for Intelligent Systems Research and Innovation, Deakin University, Australia.

Recording method: All manipulations and recordings were made in a biosafety cabinet. Each recording session consisted of 30 min of spontaneous activity recording at 0, 1, 2, 3, and 7 dpi or 15 min of each post stimulus activity recording at 8 dpi for gabazine stimulation assays. Every record started 5–10 min after placing the MEA on the amplifier to avoid neuron response to mechanical stress due to movement and to allow adaptation to the biosafety cabinet conditions and temperature set by the adaptor instead of incubator. MEAs temperature was maintained at 37 degrees Celsius through the recording. Half of the media was changed after each recording session. The raw continuous voltage traces were filtered to remove the traces of field potential below 200 Hz generated by the collectively charged network. The high pass filter was comprised of a 2nd order Butterworth filter with cutoff frequency of 200 Hz. The spike detection threshold was set at 8 times the high-pass filtered signal’s standard deviation [34] (as measured by MC_Rack software, 22 μV) within a 500 ms window.

Recording time points: Once the dissociated neurons from mouse embryos were plated in the MEA, the cultures were let to grow and maturate for 7 days. At 7 div, these primary neuron networks were infected with ZIKV or DENV2 (used as a positive control) at a MOI of 0.2.

For each experimental assay, 6 electrophysiological recordings of spontaneous activity were made. The first was done at 7 div before infection and set as reference for further analysis. After infection, records were sampled at 1, 2, 3 and 7 days post infection (dpi) (Additional file 3: Figure S1A). An additional recording was made at 2 dpi by introducing the D(−)-2-amino-5-phosphonopentanoic acid (APV) (Sigma Aldrich) at 300 μM, a NMDA-R antagonist [29]. The last recording took place at 8 dpi for stimulation with 20 μM of gabazine [35], a GABA_A_ antagonist (Tocris SR 95531 hydrobromide). Before the introduction of the APV or gabazine, a prerecording was done by adding the same volume of solvent (water) in the well as a reference for the analysis of the stimulus.

### Microelectrode Array data analysis

Microelectrode array data analysis with MC_Rack: MEA data analysis is performed offline with MC_Rack software (Multi Channel Systems, Reutlingen, Germany). Active electrodes (AE) are selected if the spike rate for the electrode is equal or higher than 0.01 spike per second. Burst electrodes are detected with the following parameters: maximum interval to start burst = 100 ms, maximum interval to end burst = 100 ms, minimum interval between bursts = 100 ms, minimum duration of burst = 10 ms, and minimum number of spikes in burst = 3 (adapted from [36], see (Additional file 3: Figure S1B). The relative total spike (TS) number changes per electrode is calculated by: Ratio = ln (TS at time point dpi)/(TS at 0 dpi) for spontaneous spike activity. For post gabazine analysis, the TS reference is changed with the activity post stimulation by solvent (water).

Microelectrode array data analysis with NeuroSigX: MC_Rack files are cut into 6 filtered files of 5 min for spontaneous activity at the different times post infection and 6 files of 2 min post gabazine stimulation at 8 dpi. Mcd files are then converted into txt files (MC_Data tool, MultiChannel System, Reutlingen, Germany) and analyzed by NeuroSigX. The NeuroSigX software uses novel spike sorting and data analysis algorithms to explore the neural spike activity and spatio-temporal behavior of the neuronal network [37, 38]. A threshold of 22 μV is employed to maintain the analytical consistency between the preliminary analysis by MC_Rack software and analysis by NeuroSigX. Raster plots, electrode activity maps and mean spike activity data and figures are extracted for temporal and spatial analysis and illustration.

### Statistical analysis and graphics

Graphs and statistical analysis are done using GraphPad Prism 5 software. All statistical tests are done using a two-tailed analysis. All statistical results are expressed with the *p*-value using the following annotations: ns for *P* > 0.05, * for *P* ≤ 0.05, ** for *P* ≤ 0.01, and *** for *P* ≤ 0.001.

## Results

### Virus impact on mouse neuron spike activity

**Fig. 1:**
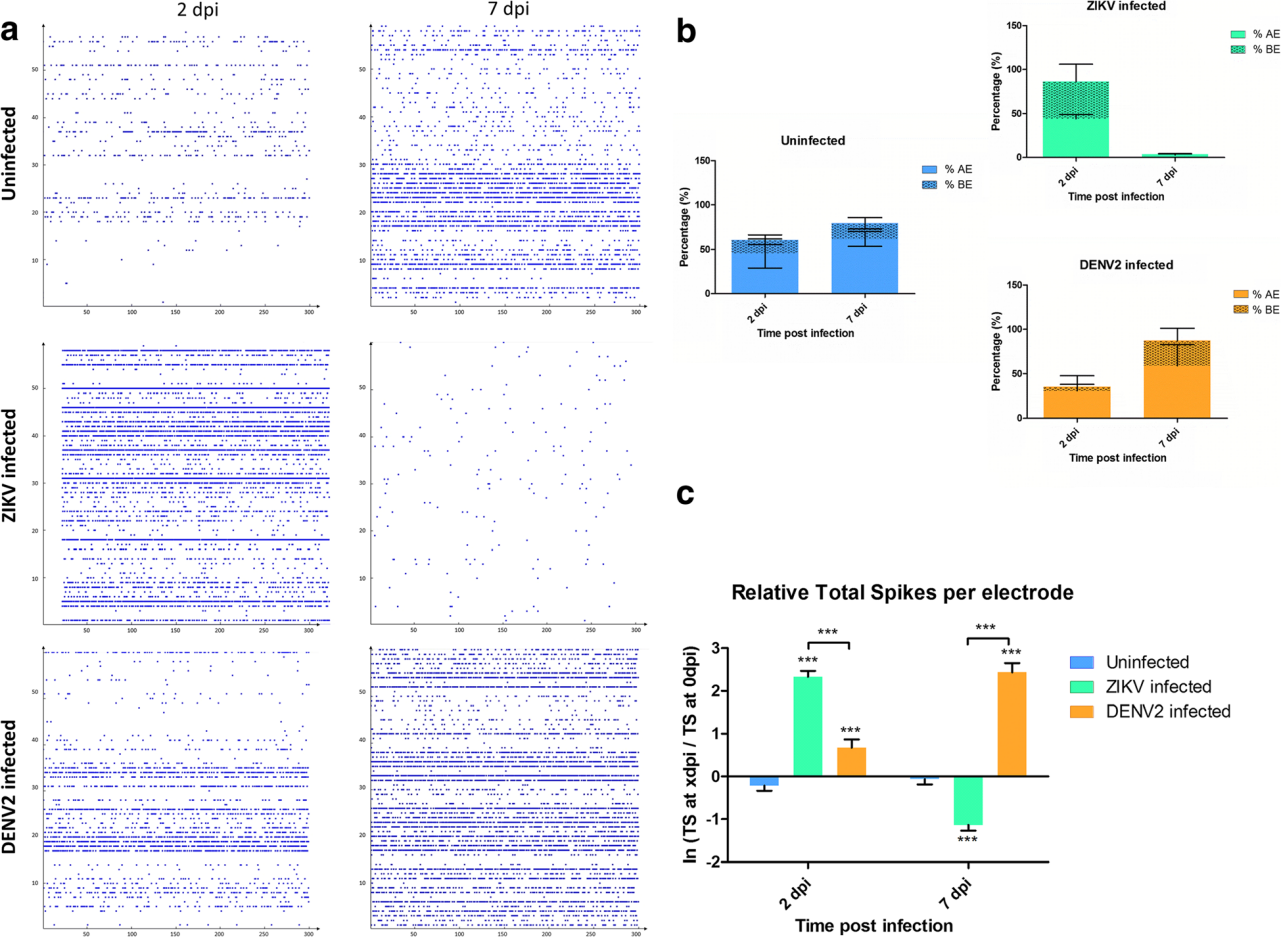
Electrophysiological analysis of mouse embryo primary neurons spontaneous activity. **a** Raster plots of 5 min spiking activity of neuronal cultures on Microelectrode array (MEA) at different days post infection (dpi). **b** Percentages of active electrodes (AE) and bursting electrodes (BE) during spontaneous neuronal activity recording at different dpi of mouse primary neuron cultures. **c** Mouse spontaneous spiking activity with average ratio of total spike (TS) number per electrode between 0 dpi (reference) and TS number at 2 and 7 dpi of spontaneous activity. Statistical differences were calculated with unpaired t tests by comparing uninfected group to infected groups unless indicated by bracket. *P < 0.05; **P < 0.01; ***P < 0.001; means ± SEM. For **c** *n* values are detailed in Additional file 7: Table S3

**Fig. 2:**
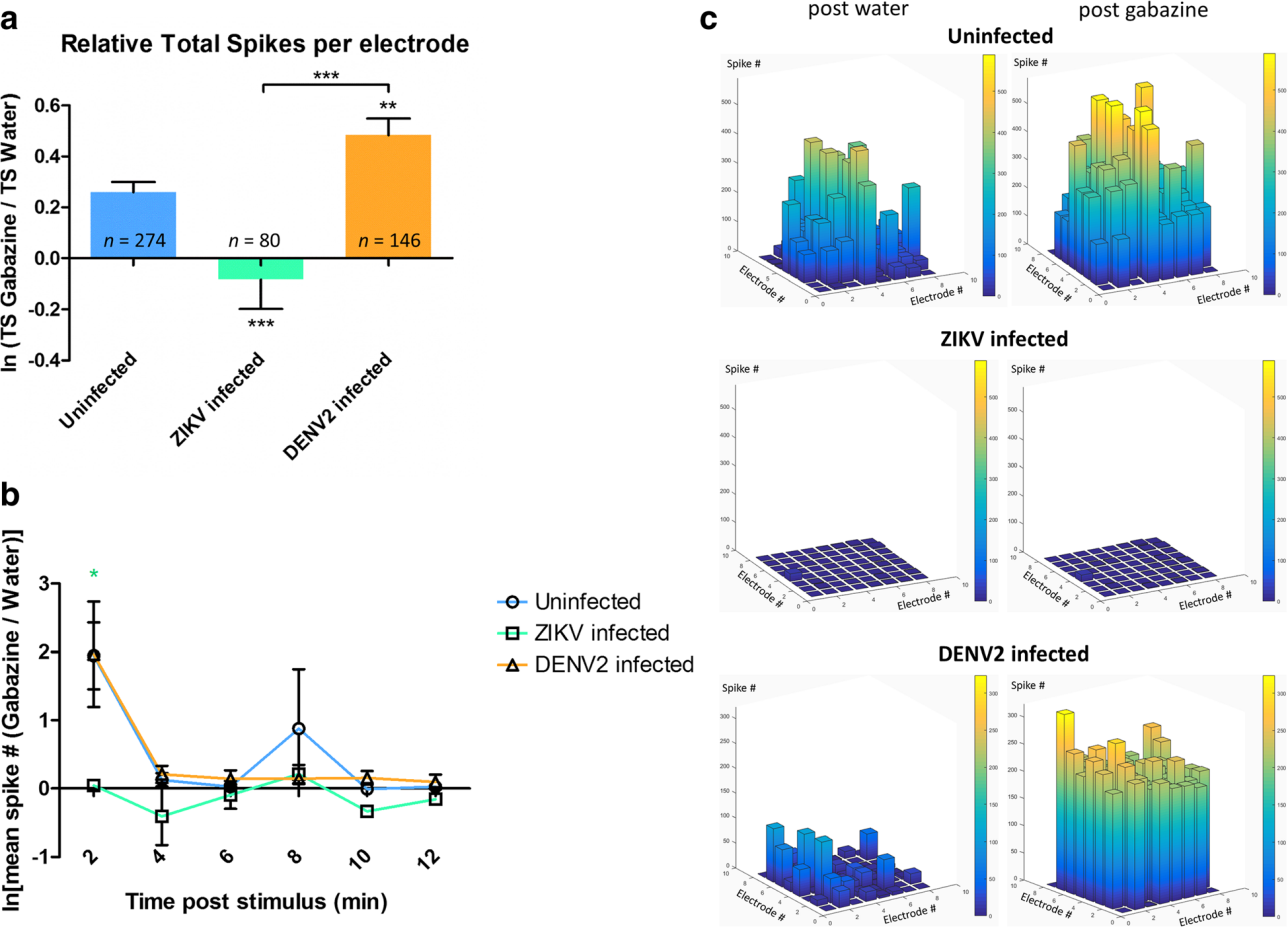
Electrophysiological analysis of mouse embryo primary neurons under gabazine stimulus at 7dpi. **a** Mouse primary neurons stimulated activity with average ratio of TS number per electrode between gabazine stimulus and solvent (water) introduction; *n* = number of active electrodes. **b** Line charts representing the average relative change of the mean spike number per electrode between the reference (TC water) and the introduction of gabazine at different time points. Statistical differences were calculated with unpaired t tests by comparing uninfected group to infected groups unless indicated by bracket. *P < 0.05; **P < 0.01; ***P < 0.001; means ± SEM. **c** 3D electrode activity maps of the effect of gabazine stimulation mouse neuronal networks for 2 min, post water as baseline and post gabazine

Given the established neurotropism of ZIKV during mammalian infection, functional impact of Zika infection in *Mus musculus* embryonic cerebral cortical cells cultured on MEA was tested. Dengue virus serotype-2 (DENV2) was used as a positive viral control. At 2 dpi, virus infections triggered hyperactivity in primary neuron cultures (Fig. 1 and Additional file 4: Figure S2A and B). At 3 dpi, the activity of infected primary neuron cultures decreased and reached an average individual electrode activity comparable to uninfected cultures (Additional file 4: Figure S2B). However, at 7 dpi, the spiking activity was significantly and dramatically decreased in ZIKV infected neuron cultures, while DENV2 infected neuron cultures remained hyperactive (Fig. 1). GABA_A_ or ɤ-Aminobutyric acid, is an amino acid inhibitory neurotransmitter widely present in the nervous system [39, 40]. In order to exclude the possibility of a GABA_A_-induced inhibitory effect, cultures from primary neuron model were treated with gabazine (SR 95531 [2-(3′-carboxy-2′-propyl)-3-amino-6-p-methoxyphenylpyridazinium bromide]), a GABA_A_ antagonist, known to remove neurotransmitter inhibition hence stimulating the firing activity of neuronal network [35]. The electrophysiological response [41, 42] was recorded in primary neurons and we found a lack of relative response to gabazine compared to solvent in cultures infected by ZIKV (Fig. 2a and b), as shown on a 3D electrodes map (Fig. 2c). Gabazine or water (solvent) was unable to provoke any response in ZIKV infected primary neurons at 7 dpi, confirming the extreme functional silencing of neuronal activity.

### Virus dynamics in mouse primary neurons

**Fig. 3:**
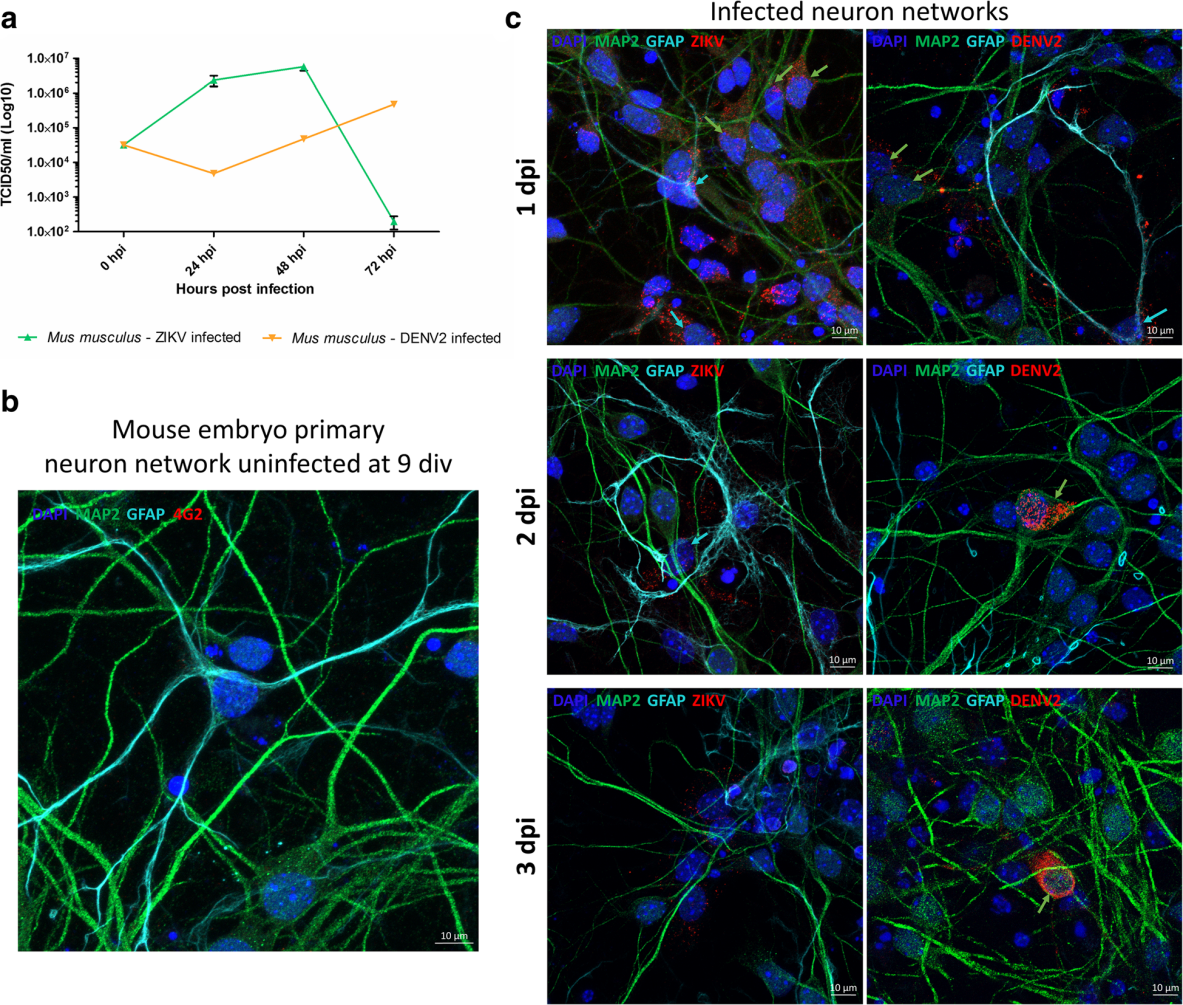
Zika virus early infection dynamics at 1, 2 and 3 dpi. **a** Zika virus replication dynamics (using TCID_50_ in Vero cells) from 0 to 72 h post infection in mouse primary neurons (*n* = 3 per condition). **b** Confocal images of an uninfected mouse embryo neuron culture at 9 div (or 2 dpi). **c** Confocal images of ZIKV (left) or DENV2 (right) infected network at 1, 2 and 3 dpi, revealing the presence of virus (4G2 in red) in neurons (MAP2 in green) and glial cells (GFAP in turquoise)

To determine ZIKV replication dynamics in mouse neurons, primary neurons from embryonic mice were infected with ZIKV and viral titers in the media supernatant were measured over time. The results showed that ZIKV replicated in primary neuron cultures until 2 dpi (Fig. 3a). However, at 3 dpi, ZIKV titer reduced dramatically by 4 logs. As control, primary neuron cultures were also infected with DENV2 (Fig. 3a). Cultures infected with DENV2 show a growth dynamic with an initial decrease followed by increase in virus titers until 3 dpi. Virus infections in the primary neuron cultures was confirmed by immunofluorescence. Uninfected cultures showed the presence of neurons with MAP2 staining and glial cells with GFAP stain (Fig. 3b). Zika virus and DENV2 staining were detected from 1 to 3 dpi in mouse cultures (Fig. 3c). DENV2 was visible mostly in neurons and in glial cells, yet at lower frequency, from 1 to 3 dpi. Zika virus was present in the cultures at 1 dpi, with signal detected in both in neurons and glial cells. The amount and frequency of ZIKV signal became lower at 2 and 3 dpi, and mostly co-localized with dense DAPI nuclei.

### Virus effects in mouse primary neuron network

**Fig. 4:**
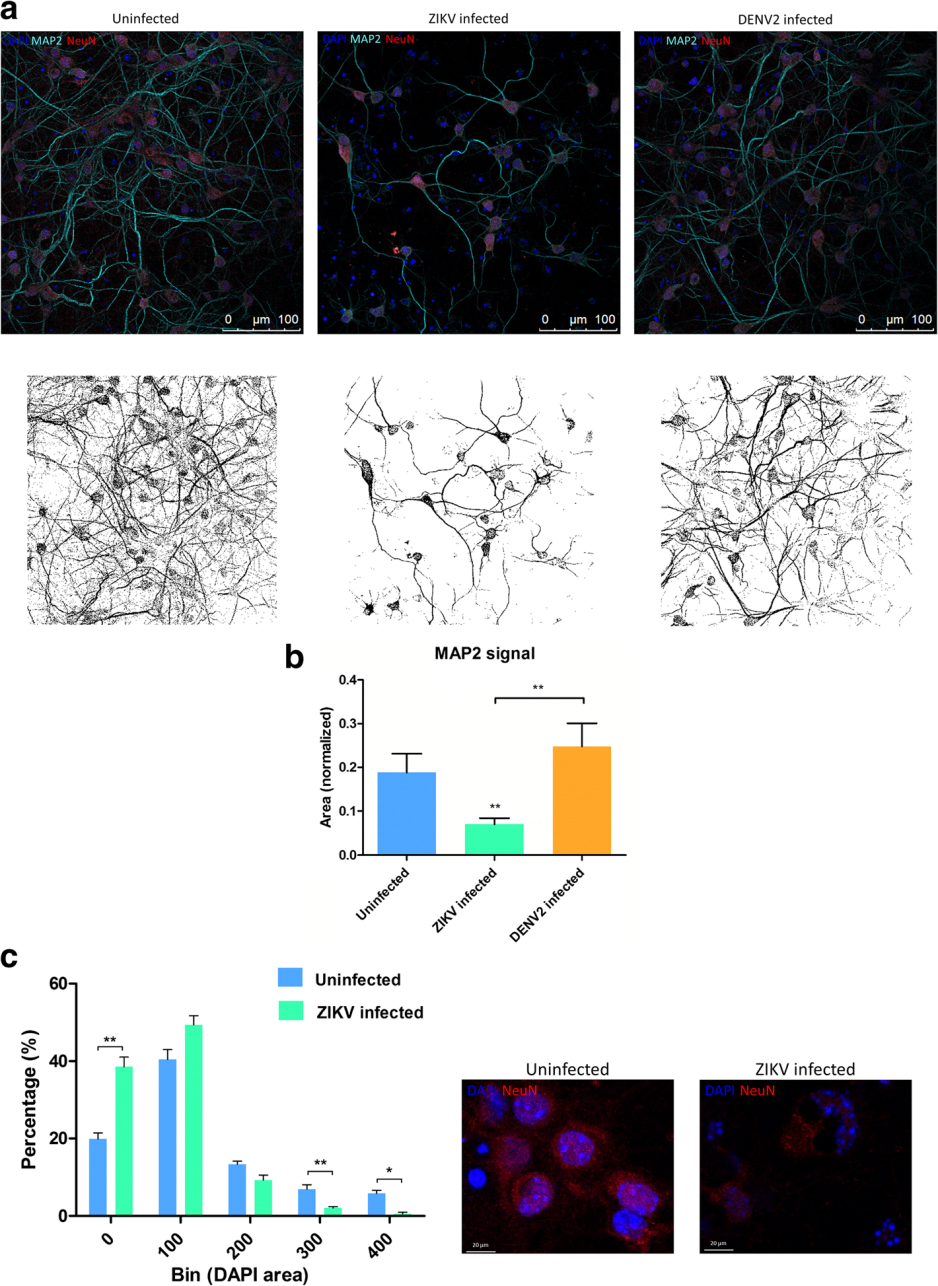
Zika virus (ZIKV) effects on primary neuron networks from at 3 dpi. **a** Representative confocal images showing DAPI marker (blue), NeuN antibody (red), and MAP2 antibody (turquoise) at the top and its corresponding images with MAP2 staining below. Image analysis with ImageJ software reveals the differences of MAP2 signaling. **b** Quantification of MAP2 coverage, normalized by the number of DAPI counts in mouse primary neuron cultures at 3 dpi, *n* values are detailed in Additional file 2: Table S2. **c** Bar plots compare the size of DAPI stained nuclei of ZIKV infected and uninfected cells at 3 dpi. Each bin represents the distribution range size of nuclei in square pixel (*n* = 5 images for each condition). Bar plot values are shown with ± Standard Error of the Mean (SEM) with Mann-Whitney *p*-value on top, *P < 0.05; **P < 0.01; ***P < 0.001. On the right, confocal pictures illustrating nuclei sizes at 3 dpi

**Fig. 5:**
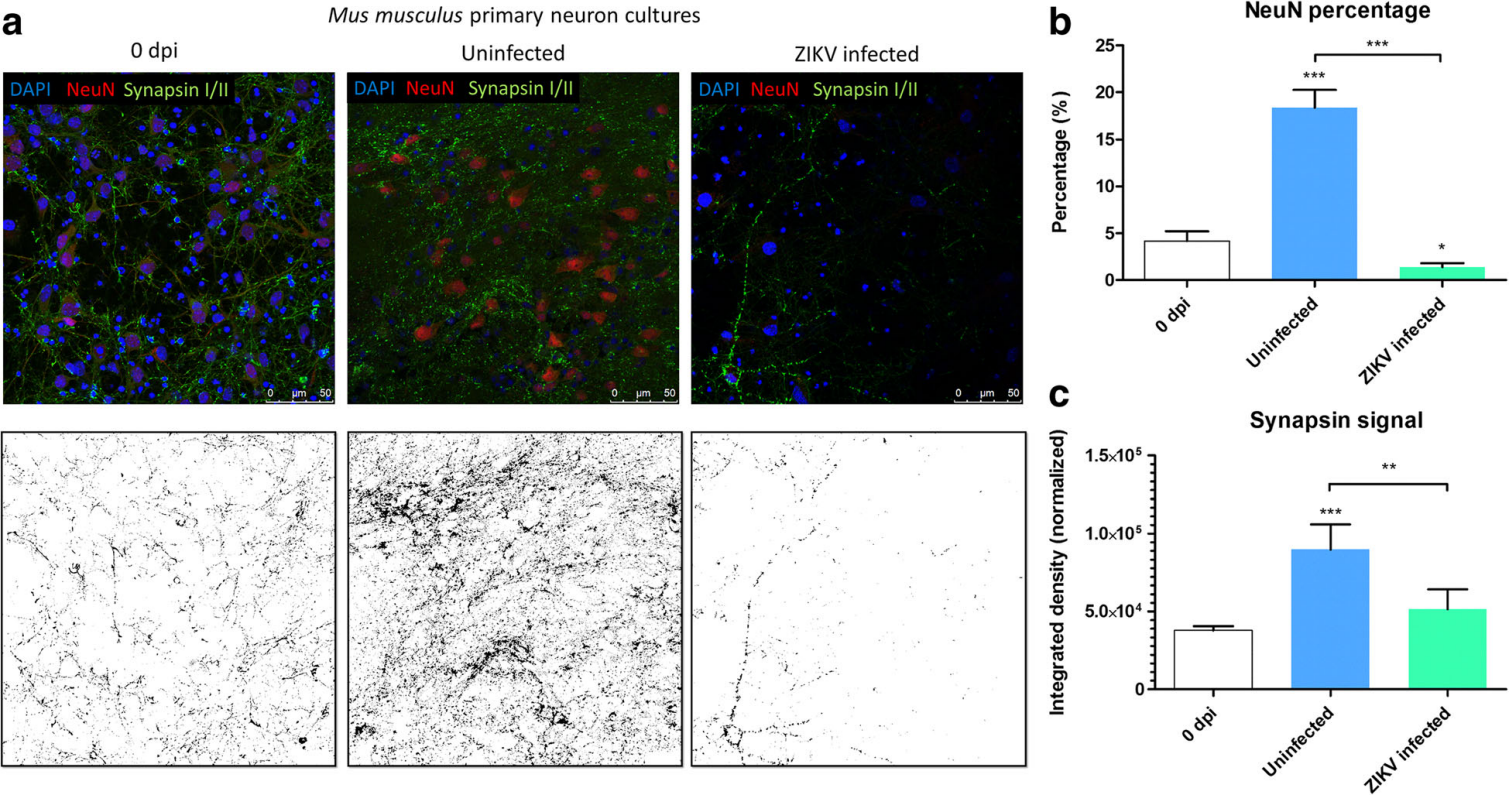
Zika virus (ZIKV) effects on primary neuron networks at 7 dpi. **a** Confocal images of mouse primary neuron culture before (0 dpi) and post ZIKV infection (7 dpi) at the top, respective Synapsin signal after applying image threshold with ImageJ at the bottom. **b** Percentage of mature mouse neurons stained with NeuN at 0 and 7 dpi, in mouse primary neuron cultures. **c** Quantification of Synapsin I/II signal, normalized by the number of DAPI counts on the right. Statistical differences were calculated with Mann-Whitney *U* tests, by comparing 0 dpi to the other groups unless indicated by bracket. *P < 0.05; **P < 0.01; ***P < 0.001; means ± SEM; *n* values are detailed in Additional file 2: Table S2

Zika virus infected neurons showed a significant decrease in the neuronal marker Microtubule Associated Protein 2 (MAP2) staining compared to the uninfected and DENV2 infected cultures (Fig. 4a and b). At the same time, ZIKV infected neuron cultures had a significant decrease in NeuN positive cells compared to uninfected cultures (Mann Whitney test, *P* = 0.008, *U* = 7.0) and DENV2 infected cultures (Mann Whitney test, *P* = 0.001, *U* = 0.0) (Additional file 4: Figure S2C). Moreover, analysis of 3 dpi ZIKV infected neuron nuclei with DAPI staining showed a significantly decreased percentage of large and heterochromatic nuclei (associated with NeuN staining of mature neurons) and an increase in small sized nuclei or fragments compared to uninfected (Fig. 4c) (Mann Whitney tests, for “bin 0” *P* = 0.0079, for “bin 300” *P* = 0.0079 and for “bin 400” *P* = 0.0112). Later during the time-course, results also showed that at 7 dpi, consistent with the functional silencing in spike detection recorded, the significant decrease in the number of matured mouse neurons in ZIKV infected cultures was verified with the NeuN, neuronal nuclear marker (Fig. 5a and b). In contrast, uninfected as well as DENV2-infected cultures showed a well-matured network with a significantly higher number of mature neurons at 7 dpi. These results confirm that, even at moderate level Multiplicity of Infection (MOI = 0.2), ZIKV infection dramatically reduced the number of mature neurons, as shown before [25].

### Neurotransmitter pathways and synapse response

**Fig. 6:**
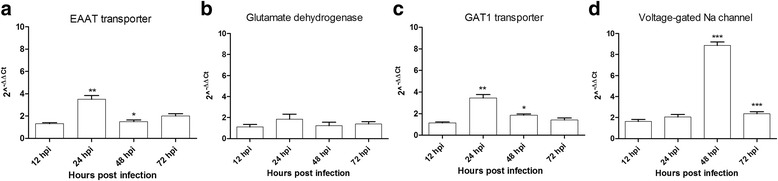
Genes mRNA expression after Zika virus infection in primary neuron cultures. Quantification of **a** Glutamate transporter (EEAT), **b** Glutamate dehydrogenase (GD1), **c** GABA_A_ transporter (GAT1) and **d** Voltage-gated sodium channel (VGNaC) mRNA expression. Statistical differences were calculated with unpaired *t*-tests by comparing mRNA expression at time_*i*_ to time_*i*-1_ within each group (*n* = 3). *P < 0.05; **P < 0.01; ***P < 0.001; means ± SEM

Our results show that the decreasing number of mouse mature neurons at 3 dpi is preceded by neuronal spiking hyperactivity (Additional file 4: Figure S2C). In mammals, glutamate is the main excitatory neurotransmitter [43]. A recent report demonstrates the efficacy of memantine, a blocker of NMDA-R, in decreasing Zika-induced neurodegeneration in the brain of infected mice, implying a possible toxic role of the excitatory neurotransmitter glutamate [25]. Real time RT-PCR results showed an early significant increase in the mRNA expression of EAAT (Excitatory Amino Acid Transporter, a glutamate transporter at post-synaptic level) at 24 h post infection (hpi), but returned to baseline level at 2 dpi (Fig. 6a). On the other hand, mRNAs of the Glutamate Dehydrogenase (GD1), a mitochondrial enzyme responsible for glutamate oxidation and recycling towards the tricarboxylic cycle by mouse astrocytes [44] (Fig. 6b), as well as the VGlut (Vesicular Glutamate Transporter, a cytoplasmic pre-synaptic glutamate transporter) showed no substantial change over time after infection in primary neurons (Additional file 5: Figure S3). Regarding the GABA_A_ pathway, pre-synaptic GABA_A_ transporter 1 (GAT1) mRNA was significantly over-expressed only transiently in ZIKV infected neurons at 24 hpi (Fig. 6c) while the expression profile of the post-synaptic GABA receptor remained unchanged over time (Additional file 5: Figure S3). Finally, the Voltage-gated sodium channel (VGNaC), responsible for the propagation of action potential, mRNA expression increased in mouse neurons with a peak at 48 hpi (Fig. 6d), corresponding to the hyper-excitatory phase recorded with MEA (Fig. 1). These results indicate that ZIKV infection leads to differential gene expression of glutamate and GABA pathway genes in mouse primary neurons at early stage of infection with ZIKV. The early and transient upregulation of EAAT could decrease the synaptic concentration of the excitatory neurotransmitter glutamate by increase of its transporter. As well, the brief upregulation of GAT1 may improve the presynaptic availability of the inhibitory neurotransmitter GABA. However, these gene regulation are not enough to compensate the long-lasting excitation post infection and first detrimental effects are observed at 3 dpi (Figs. 3 and 5).

Immunofluorescence result showed that at 7 dpi, there was a decrease of the phosphoprotein Synapsin I/II signal in ZIKV infected neuron networks compared with uninfected control (Fig. 5a and c). Along with mRNA data, the decrease in Synapsin protein in mouse primary neurons after ZIKV infection confirms the importance of this protein in neuronal networks and its potential role in neuronal damage mechanisms post infection, as it has been shown to accelerate synaptic vesicle traffic during repetitive stimulation [45]. Except the EAAT and GAT1 transient and potentially protective regulations, the absence of durable regulatory mechanisms in mouse neural tissue to offset the excitatory effect of glutamate, notably the absence of upregulation of GD1 for recycling glutamate by astrocytes [46], could explain the extensive neuronal death [25], over and above the viral cytopathogenic effect.

### Implication of N-methyl-D aspartic acid receptors during Zika virus infection

**Fig. 7:**
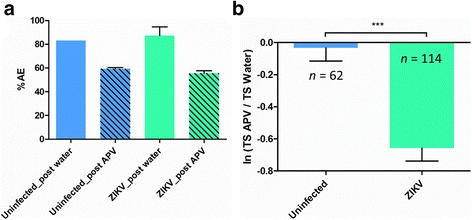
Microelectrode array (MEA) recording at 2 dpi after a NMDA-R antagonist introduction in mouse primary neuron cultures. **a** Percentage of Active Electrodes (AE) average from neuron network cultures after introduction of the NMDA-R antagonist APV (*n*_MEA_ = 3 per condition). **b** Electrode activity calculated as average ratio of Total Spike (TS) number per electrode between APV and solvent introduction; Mann-Whitney *U* test, *P < 0.05; **P < 0.01; ***P < 0.001; means ± SEM; *n* = number of active electrodes

To test the hypothesis that the hyper excitation observed in ZIKV infected mouse neurons (Fig. 1) was due to an increase of glutamate neurotransmitter, NMDA-R antagonist, APV, was introduced to the mouse neuron cultures on MEA at 2 dpi. The analysis of the electric activity post APV introduction was compared to the neuron activity after introduction of the solvent (water). The antagonist caused a decrease of the network activity with 20% less AE for uninfected and ZIKV infected cultures (Fig. 7a). However, uninfected neuron cultures had a significantly lesser decrease of individual spike activity compared to ZIKV infected neuron cultures with ln(TS_APV_/TS_Water_) = − 0.034, ± 0.08 SEM for uninfected cultures and ln(TS_APV_/TS_Water_) = − 0.66, ± 0.08 SEM for ZIKV infected cultures (Kruskal-Wallis test, *P* = 0.019) (Fig. 7b). These results indicate that the electrode hyper activity observed at 2 dpi in ZIKV infected cultures was mostly due to the excitation of NMDA-R and glutamate neurotransmitter.

## Discussion

Since the last outbreak in South America, ZIKV infections are linked to neurological disorders, such as microcephaly and GBS. Although this is major public health concern, there are no approved vaccines or efficient therapies available. Many questions remain regarding pathogenesis mechanisms by which ZIKV is infecting the CNS as well as the PNS. Our study uses a mouse primary neuron culture model for infection with ZIKV to study its functional impact on neuronal network. Extensively used for toxicology assays, rodent primary neurons on MEA gives the possibility to monitor short and long term effects of the drugs on primary neuron culture electrophysiology [47]. The culture of primary neurons on MEA provides the possibility to record electrical signal from the neuronal network, which represents a more biologically relevant system (including neurons and glial cells) than a one-type cell culture. Indeed, in the primary culture used in this study, there are also glial cells along with mature neurons (Fig. 3b and Additional file 6: Figure S4). By using this technique, our results show that ZIKV triggers hyperactivity in mouse neuronal networks during the early stage of infection. Positive control neuron cultures infected with DENV2 also display an increase of neuronal spike activity (Fig. 1c). However, this electrical hyper excitation is followed by an almost complete silencing of electrical activity in ZIKV infected neuronal networks at 7 dpi. This lack of activity is confirmed by the non-response of the network post-gabazine stimulus, where it triggers an increase of activity in uninfected and DENV2 infected cultures (Fig. 2a and b). Our results also show neuronal loss confirmed by confocal microscopy with a significant decrease in number of mature neurons (Fig. 5c and Additional file 3: Figure S1C) as well as network density (Fig. 4a and b), and condensed small nuclei (Fig. 4c) in ZIKV infected cultures. Neuronal death of neural progenitor cells and differentiated neurons, was previously reported as virally induced apoptosis and cell-cycle dysregulation [6, 23].

Overstimulation by a neurotransmitter, most frequently glutamate, has been shown to be neurotoxic at high concentrations to mammalian neurons, with damage or death of neighboring neurons connected by glutamatergic synapses, due to metabolic deficit [43, 48]. Neuron activity, recorded at the time of maximum hyperactivity of ZIKV infected cultures, *i. e*. 2 dpi, showed a significant decrease of spike activity after the introduction of a NMDA-R antagonist (Fig. 7a and b). This confirmed that the spontaneous hyper excitation observed (Fig. 1) was triggered via the glutamate pathway. Our results are compatible with the Costa et al. hypothesis, that blocking NMDARs with antagonists could provide potent neuroprotective effects against ZIKV-induced neuronal damage [25]. Given the low infective virus load (MOI = 0.2) used in this study and low virus replication dynamics, excitotoxicity may be partly responsible for the observed massive decrease in the number of mature mouse neurons, consistent with results by Costa et al. [25], by magnifying the virus-induced cytopathogenic effect and associated deleterious immune responses. Interestingly, our results show that the virus tends to be cleared out from the mouse neuron cultures from 3 dpi (Fig. 3a and c). Although not complete clearance, similar decrease of titer at 4 dpi was observed by Costa et al. [25] using a higher dose of virus infection (MOI = 1). This trend of decreased virus titer may characterize a pattern of resistance to the pathogen at the organism level but in turn is detrimental to neuronal health [49]. This lethal mechanism via glutamate was not observed with our positive control with DENV2, although the virus is closely related to ZIKV. Electrical recording also showed a hyper excitation of DENV2 infected neuronal network but no significant mature neuron decrease has been observed.

We found that ZIKV infected primary cultures have an increase expression of some key genes in neuronal communication, such as VGNaC, GAT1 and EEAT. During cerebral ischemia in humans, the increase of EAAT expression is protective [50]. In human cases of West Nile virus-induced acute flaccid paralysis, and in related hamster model, the EEAT expression is decreased in the spinal grey matter [51], demonstrating its association with protection against neuronal damage. The early and transient upregulation of EAAT could decrease the synaptic concentration of the excitatory neurotransmitter glutamate by increase of its transporter. As well, the brief upregulation of GAT1 may improve the presynaptic availability of the inhibitory neurotransmitter GABA. However, these gene regulations are not enough to compensate the long-lasting excitation post infection and first detrimental effects are observed at 3 dpi.

Our model includes neurons but also glial cells, especially astrocytes, where the presence of ZIKV has been confirmed as soon as 1 dpi in our infected cultures (Fig. 3c). Recent studies have also demonstrated the implication of glial cells in ZIKV infection. Li et al. found replicating virus in mature neurons as well as astrocytes [22]. Another study found that oligodendrocytes – also glial cells - were more susceptible to ZIKV than neurons [52]. Finally, after infection of newborn mice, astrocytes have been found to be the first cell type to be infected by ZIKV [53]. Astrocytes have pivotal and multiple roles in the homeostasis of neuronal network, at the crossroad of regulation of metabolism, immune response and synaptic activity modulation [54]. These findings suggest a key role of glial cells and the importance of the neuronal network during ZIKV infection of CNS and PNS. Glutamate Dehydrogenase 1, mostly found in astrocytes and responsible for glutamate degradation, is not upregulated in ZIKV infected cultures, which could be due to a lack of defense mechanisms after increase of glutamate or to the absence of astrocytes in the network due to cell death.

## Conclusions

In conclusion, the present study shows that in vitro ZIKV infection of mouse primary neuron networks triggers excitotoxicity leading to neuronal loss and decrease of synapses. The use of MEA to record neuronal electrical activity after viral infection provides new evidence and insight about ZIKV pathogenesis mechanisms and its consequences on neuronal networks. Our results also showed that ZIKV and DENV2, another flavivirus, infections prompt different fates for neurons, validating the uniqueness of ZIKV infections in mammalians nervous systems. This study provides a possible therapeutic target to reduce the impact of Zika virus infection.

## Additional files

Additional file 1: **Table S1.** Primers table for mouse (*Mus musculus*) genes used for Q-PCR. (PDF 88 kb)

Additional file 2: **Table S2.** Summary table of *n* used for statistical data analysis of confocal images. (PDF 92 kb)

Additional file 3: **Figure S1.** Experimental procedure of primary neuron culture recording on microelectrodes array. (PDF 287 kb)

Additional file 4: **Figure S2.** Temporal analysis of *Mus musculus* neuronal networks spontaneous activity on Microelectrode array post infection. (PDF 181 kb)

Additional file 5: **Figure S3.** Gene expression comparison after Zika virus infection in neuron cultures. (PDF 297 kb)

Additional file 6: **Figure S4.** Mouse primary neuron culture at 14 days post seeding. (PDF 222 kb)

Additional file 7: **Table S3.** Summary table of statistical data analysis of mouse neuron cultures spontaneous activity at different times post infection (Fig. 1c). (PDF 36 kb)

## Abbreviations

AE: Active electrode
APV: (2*R*)-amino-5-phosphonovaleric acid; (2*R*)-amino-5-phosphonopentanoate
BE: Bursting electrode
CNS: Central nervous system
DENV2: Dengue virus serotype-2
dpi: days post infection
EAAT: Excitatory Amino Acid Transporter
GAT1: GABA_A_ transporter 1
GBS: Guillain-Barre syndrome
GD1: Glutamate Dehydrogenase
hpi: hours post infection
MEA: Microelectrode array
PNS: Peripheral nervous system
TS: Total spike
VGlut: Vesicular Glutamate Transporter
VGNaC: Voltage-gated sodium channel
ZIKV: Zika virus
