# Supplementary material for: Zika virus-induced hyper excitation precedes death of mouse primary neuron
Source: Virol J. 2018 Apr 27;15:79. doi: 10.1186/s12985-018-0989-4 (PMC5922018; doi:10.1186/s12985-018-0989-4)
Supplement: Supplementary file 1 — Table S1. Primers table for mouse (Mus musculus) genes used for Q-PCR. (PDF 88 kb) [file 12985_2018_989_MOESM1_ESM.pdf]

Supplementary Table 1. Forward (grey background) and reverse (white background) primers for mouse (*Mus musculus*) genes used for Q-PCR.

Mus musculus (mouse)	
**Voltage-gated Na channel type II (NM_001099298)**	CTTGCAAGGGGCTTTTGTCTA
	GGTTTACAAATTCTGTTACATACGC
**Glutamate dehydrogenase 1 (NM_008133)**	AGCTGGCCAAGAAGGGTTTT
	GCATAGGTGTCAGCGATCCA
**EAAT3 (NM_009199)**	GAAAAGCCAGGCCATAAGCG
	AGCCACAAGGCTGAGATTCC
**vGlut (NM_080853)**	CGGGAGCGGTCAGGTTTTAT
	GCTGATCTTTGCGAACGTGA
**GAT1 (NM_178703)**	CCTACACTGGCACTCTGGAC
	GGTCATGGTGTCTGGACCTG
**GABA receptor subunit (NM_008069)**	TTGGGGCTTCTCTCTTTTCCC
	GTCGGTCCACTGTCTCTTTCA
**18s rRNA**	GTAACCCGTTGAACCCCATT
	CCATCCAATCGGTAGTAGCG
