# Supplementary material for: Zika virus-induced hyper excitation precedes death of mouse primary neuron
Source: Virol J. 2018 Apr 27;15:79. doi: 10.1186/s12985-018-0989-4 (PMC5922018; doi:10.1186/s12985-018-0989-4)
Supplement: Supplementary file 2 — Table S2. Summary table of n used for statistical data analysis of confocal images. (PDF 92 kb) [file 12985_2018_989_MOESM2_ESM.pdf]

Supplementary Table 2. Summary of *n* used for statistical data analysis of confocal images.

		Fig. 5b	Fig. 5c	Fig. 4b
n, images	**0 dpi**	-	8	7
	**Uninfected**	8	18	13
	**ZIKV infected**	7	11	12
	**DENV2 infected**	6	10	-
n, total cells number	**0 dpi**	-	1845	333
	**Uninfected**	1294	2366	565
	**ZIKV infected**	878	761	565
	**DENV2 infected**	770	1725	-
n, coverslips	**0 dpi**	-	2	2
	**Uninfected**	2	3	3
	**ZIKV infected**	2	4	3
	**DENV2 infected**	2	4	-
n, experimental repeats	**0 dpi**	-	1	1
	**Uninfected**	1	1	2
	**ZIKV infected**	1	1	2
	**DENV2 infected**	1	-	-
