# Supplementary material for: Zika virus-induced hyper excitation precedes death of mouse primary neuron
Source: Virol J. 2018 Apr 27;15:79. doi: 10.1186/s12985-018-0989-4 (PMC5922018; doi:10.1186/s12985-018-0989-4)
Supplement: Supplementary file 3 — Figure S1. Experimental procedure of primary neuron culture recording on microelectrodes array. (PDF 287 kb) [file 12985_2018_989_MOESM3_ESM.pdf]

**A**

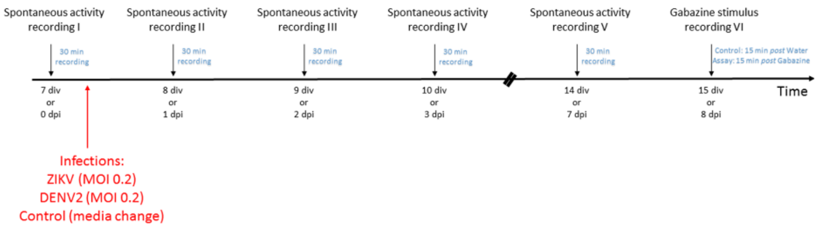


**B**

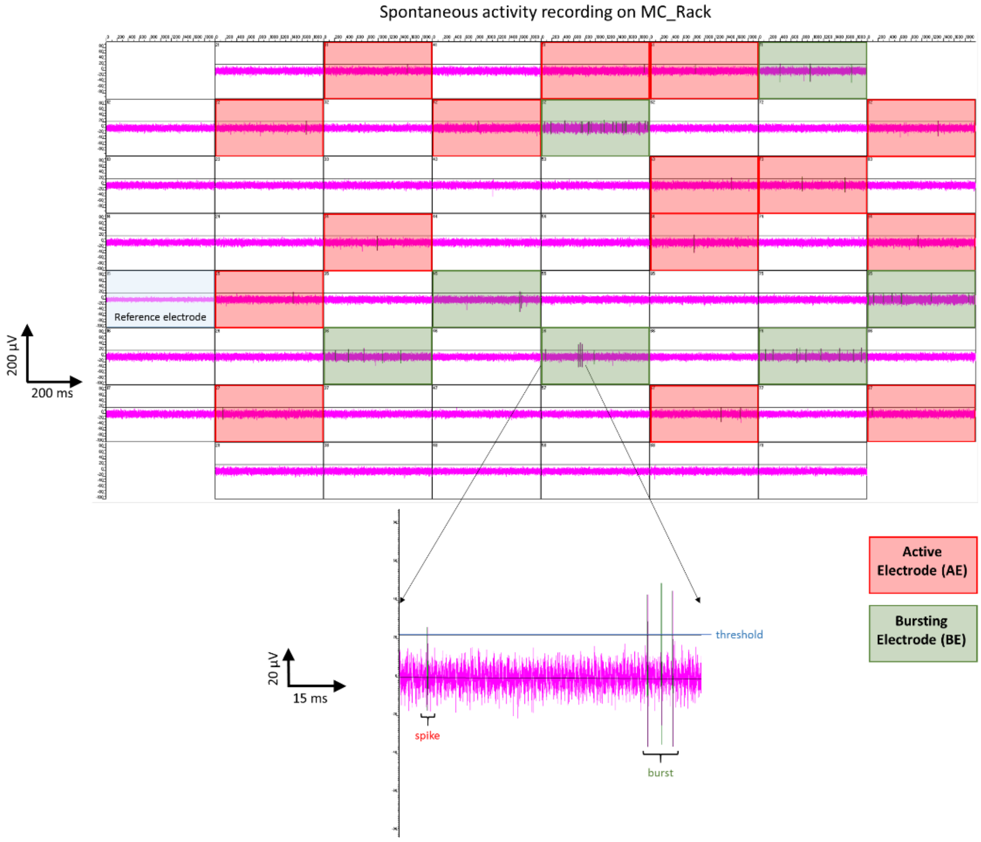


**Supplementary Figure 1. Procedure of primary neuron culture recording on microelectrodes array (MEA). A** Infection and temporal recording of primary neuron networks on MEA. **B** Experimental setup and MEA analysis parameters illustration.
