# Supplementary material for: Zika virus-induced hyper excitation precedes death of mouse primary neuron
Source: Virol J. 2018 Apr 27;15:79. doi: 10.1186/s12985-018-0989-4 (PMC5922018; doi:10.1186/s12985-018-0989-4)
Supplement: Supplementary file 4 — Figure S2. Temporal analysis of Mus musculus neuronal networks spontaneous activity on Microelectrode array post infection. (PDF 181 kb) [file 12985_2018_989_MOESM4_ESM.pdf]

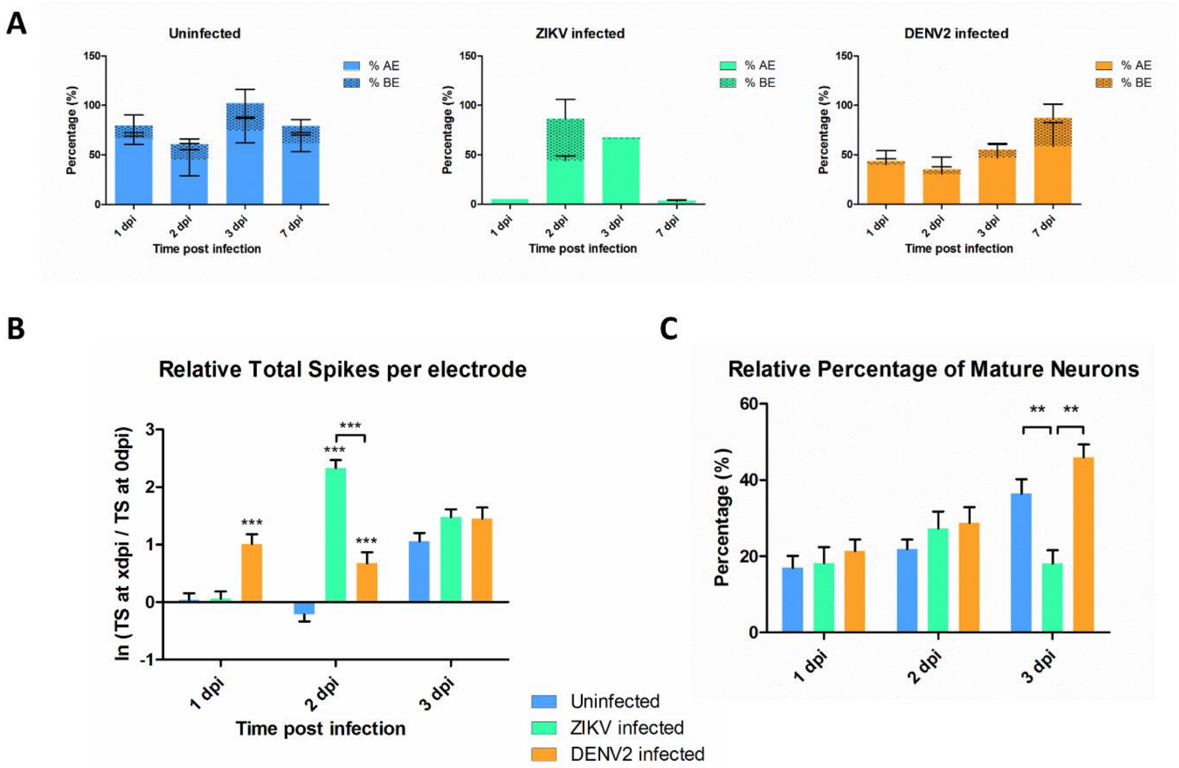

Supplementary Figure 2. Temporal analysis of *Mus musculus* neuronal networks spontaneous activity on Microelectrode array post infection. **A** Mouse cultures bar plots representing the average proportion of Active Electrodes (AE) and Bursting electrodes (BE) per MEA for each group at different time points post ZIKV and DENV2 infections. **B** Bar plots representing the average ratio of total spike (TS) number per electrode between 0 dpi (reference) and TS number at 1, 2, and 3 of spontaneous activity. All bar plots show mean value with ±SEM and statistical tests *p*-value are indicated on top of bars when compared to uninfected value. *T*-test results are summarized in Supplementary Table 1. **C** Bar plots representing the average percentage of mature neurons on the culture (percentage calculated at 1, 2 and 3 dpi. All bar plots show mean value with ±SEM and statistical tests *p*-value are indicated on top brackets for Mann Whitney tests. *$P < 0.05$; **$P < 0.01$; ***$P < 0.001$.
