# Supplementary material for: Zika virus-induced hyper excitation precedes death of mouse primary neuron
Source: Virol J. 2018 Apr 27;15:79. doi: 10.1186/s12985-018-0989-4 (PMC5922018; doi:10.1186/s12985-018-0989-4)
Supplement: Supplementary file 5 — Figure S3. Gene expression comparison after Zika virus infection in neuron cultures. (PDF 297 kb) [file 12985_2018_989_MOESM5_ESM.pdf]

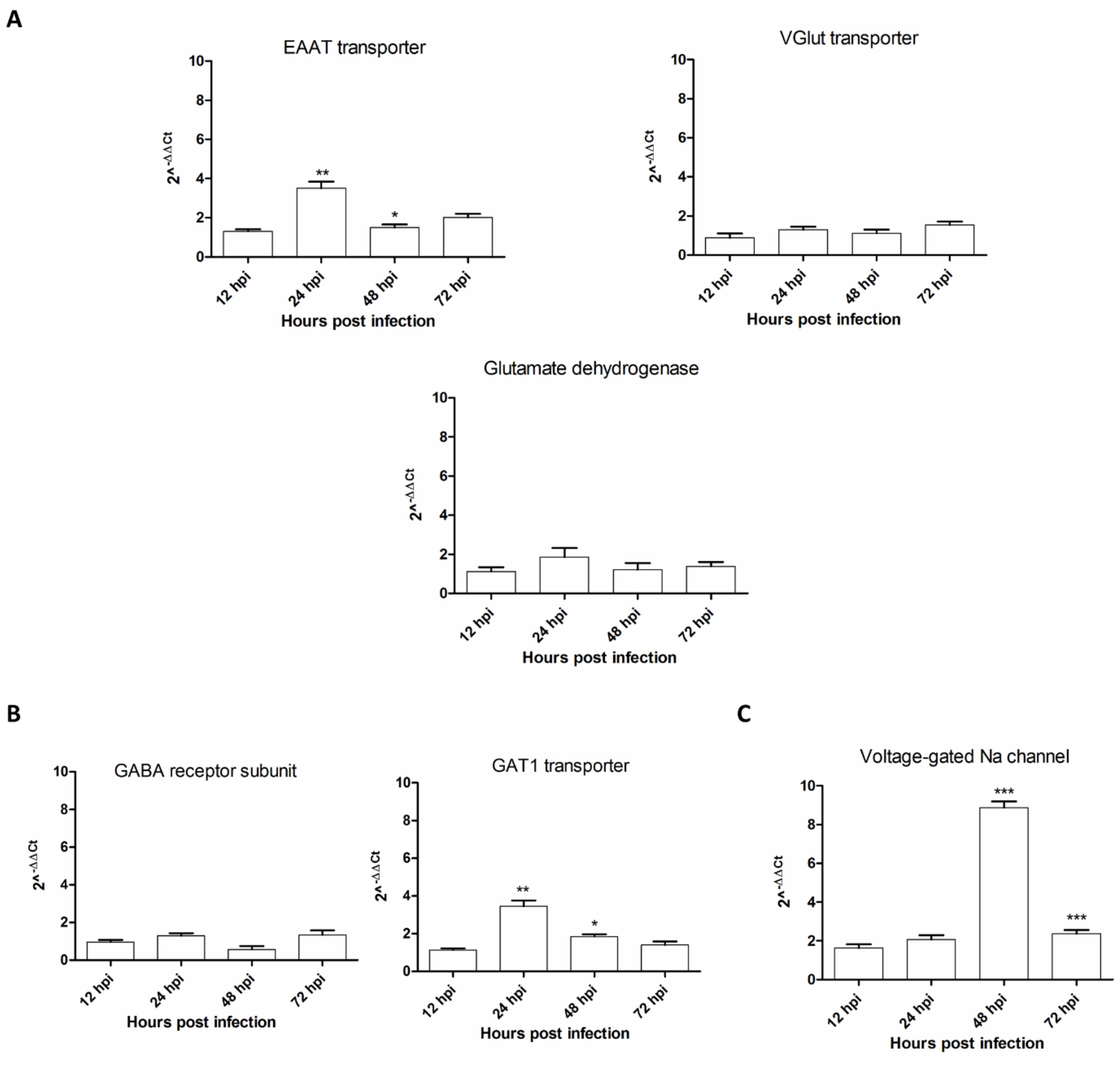

Supplementary Figure 3. Gene expression comparison after ZIKV infection in neuron cultures. *Mus musculus* neuron culture were infected with ZIKV and real-time PCR was performed at various time points. **A** Bar plots illustrating mRNA expression of Glutamate pathway proteins: Glutamate transporters (EEAT and VGlut) and Glutamate dehydrogenase (GD1). **B** Bar plots illustrating mRNA expression of $GABA_A$ pathway proteins: $GABA_A$ receptor subunit and $GABA_A$ transporter (GAT1). **C** Bar plots illustrating mRNA expression of Voltage-gated sodium channel (VGNaC). All bar plots show mean value with ±SEM (*n* = 3) of fold increase compared both to housekeeping gene and uninfected culture (ratio of $2^{-\Delta\Delta Ct}$) at the same time point in hours post infection (hpi). Statistical differences were calculated with unpaired t tests by comparing mRNA expression at $time_i$ to $time_{i-1}$ within each group.
