# Supplementary material for: Zika virus-induced hyper excitation precedes death of mouse primary neuron
Source: Virol J. 2018 Apr 27;15:79. doi: 10.1186/s12985-018-0989-4 (PMC5922018; doi:10.1186/s12985-018-0989-4)
Supplement: Supplementary file 6 — Figure S4. Mouse primary neuron culture at 14 days post seeding. (PDF 222 kb) [file 12985_2018_989_MOESM6_ESM.pdf]

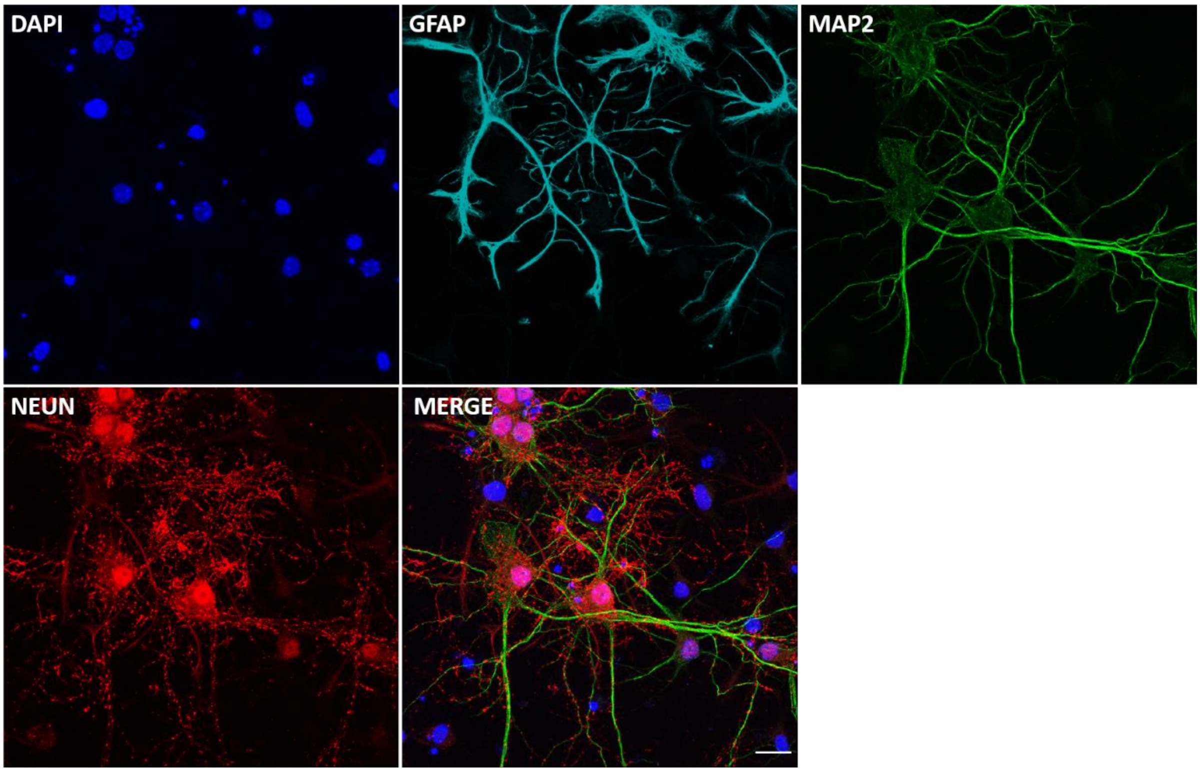

Supplementary Figure 4. Mouse primary neuron culture at 14 days post seeding. *Mus musculus* primary neuron culture observed under confocal microscopy with different staining for neuronal network structure. GFAP staining reveals the presence of glial cells in the culture. Scale bar is 20 μm.
