# Supplementary material for: Zika virus-induced hyper excitation precedes death of mouse primary neuron
Source: Virol J. 2018 Apr 27;15:79. doi: 10.1186/s12985-018-0989-4 (PMC5922018; doi:10.1186/s12985-018-0989-4)
Supplement: Supplementary file 7 — Table S3. Summary table of statistical data analysis of mouse neuron cultures spontaneous activity at different times post infection (Fig. 1c). (PDF 36 kb) [file 12985_2018_989_MOESM7_ESM.pdf]

Table S3. Summary of statistical data analysis of mouse neuron cultures spontaneous activity at different times post infection (Fig. 1c).

	Mouse (*Mus musculus*)			
**Time line**	1 dpi	2 dpi	3 dpi	7 dpi
**Uninfected to ZIKV**	t = 0.078,<br>df = 217<br>$n_{uninfected}$ = 164<br>n_{ZIKV} = 55	t = 8.317,<br>df = 452<br>$n_{uninfected}$ = 281<br>n_{ZIKV} = 173	t = 1.816,<br>df = 217<br>$n_{uninfected}$ = 164<br>n_{ZIKV} = 55	t = 5.65,<br>df = 451<br>$n_{uninfected}$ = 281<br>n_{ZIKV} = 172
	NS	$P < 0.0001$	NS	$P < 0.0001$
**Uninfected to DENV2**	t = 4.903,<br>df = 299<br>$n_{uninfected}$ = 164<br>n_{DENV2} = 137	t = 4.735,<br>df = 426<br>$n_{uninfected}$ = 281<br>n_{DENV2} = 147	t = 1.897,<br>df = 298<br>$n_{uninfected}$ = 164<br>n_{DENV2} = 136	t = 10,<br>df = 419<br>$n_{uninfected}$ = 281<br>n_{DENV2} = 140
	$P < 0.0001$	$P < 0.0001$	NS	$P < 0.0001$
**ZIKV to DENV2**	t = 3.419,<br>df = 190<br>n_{ZIKV} = 55<br>n_{DENV2} = 137	t = 3.553,<br>df = 318<br>n_{ZIKV} = 173<br>n_{DENV2} = 147	t = 0.079,<br>df = 189<br>n_{ZIKV} = 55<br>n_{DENV2} = 136	t = 14,<br>df = 310<br>n_{ZIKV} = 172<br>n_{DENV2} = 140
	$P = 0.0008$	$P = 0.0004$	NS	$P < 0.0001$
